# Exploring the gonad transcriptome of two extreme male pigs with RNA-seq

**DOI:** 10.1186/1471-2164-12-552

**Published:** 2011-11-08

**Authors:** Anna Esteve-Codina, Robert Kofler, Nicola Palmieri, Giovanni Bussotti, Cedric Notredame, Miguel Pérez-Enciso

**Affiliations:** 1 Departament de Ciència Animal i dels Aliments, Universitat Autònoma de Barcelona, 08193 Bellaterra, Spain; 2 Center for Research in Agricultural Genomics (CRAG), Campus UAB, 08193 Bellaterra, Spain; 3 Institut für Populationsgenetik, Vetmeduni Vienna, Veterinärplatz 1, 1210 Vienna, Austria; 4 Bioinformatics and Genomics, Centre for Genomic Regulation (CRG) and Universitat Pompeu Fabra (UPF), Carrer del Doctor Aiguader 88, Barcelona, Spain; 5 Institut Català de Recerca i Estudis Avançats (ICREA), Carrer de Lluís Companys 23, 08010 Barcelona, Spain

## Abstract

**Background:**

Although RNA-seq greatly advances our understanding of complex transcriptome landscapes, such as those found in mammals, complete RNA-seq studies in livestock and in particular in the pig are still lacking. Here, we used high-throughput RNA sequencing to gain insight into the characterization of the poly-A RNA fraction expressed in pig male gonads. An expression analysis comparing different mapping approaches and detection of allele specific expression is also discussed in this study.

**Results:**

By sequencing testicle mRNA of two phenotypically extreme pigs, one Iberian and one Large White, we identified hundreds of unannotated protein-coding genes (PcGs) in intergenic regions, some of them presenting orthology with closely related species. Interestingly, we also detected 2047 putative long non-coding RNA (lncRNA), including 469 with human homologues. Two methods, DEGseq and Cufflinks, were used for analyzing expression. DEGseq identified 15% less expressed genes than Cufflinks, because DEGseq utilizes only unambiguously mapped reads. Moreover, a large fraction of the transcriptome is made up of transposable elements (14500 elements encountered), as has been reported in previous studies. Gene expression results between microarray and RNA-seq technologies were relatively well correlated (r = 0.71 across individuals). Differentially expressed genes between Large White and Iberian showed a significant overrepresentation of gamete production and lipid metabolism gene ontology categories. Finally, allelic imbalance was detected in ~ 4% of heterozygous sites.

**Conclusions:**

RNA-seq is a powerful tool to gain insight into complex transcriptomes. In addition to uncovering many unnanotated genes, our study allowed us to determine that a considerable fraction is made up of long non-coding transcripts and transposable elements. Their biological roles remain to be determined in future studies. In terms of differences in expression between Large White and Iberian pigs, these were largest for genes involved in spermatogenesis and lipid metabolism, which is consistent with phenotypic extreme differences in prolificacy and fat deposition between these two breeds.

## Background

Understanding the mammal transcriptome architecture has proven to be a complex task [1-4]. The advent of high throughput sequencing technologies, such as RNA-seq, has, yet, substantially improved our comprehension of its structure and expression patterns. By deep sequencing the poly-A RNA fraction, it is possible not only to better characterize isoforms from known genes (e.g., identifying novel exons, new transcription start sites and alternative polyadenylation sites), but also to improve the annotation by discovering novel predicted coding genes and polyadenylated processed transcripts such as long intergenic non-coding RNAs [5]. Although several surveys of the transcriptome from different tissues have been conducted in humans and model species [6-17] our knowledge of livestock species remains limited. For instance, the relation between extreme phenotypic differences and their transcriptome patterns is poorly studied. The transcriptome of livestock species is, by comparison to model species, much less known despite its economic and social interest.

In this study, we used high-throughput transcriptome sequencing in two pigs from extreme breeds. Our aim was to discover and characterize novel expressed transcripts and to identify differentially expressed genes that may explain some of the phenotypic variation. We sequenced the male gonad transcriptome of a Large White and an Iberian pig, two highly divergent phenotypic breeds in terms of production traits, e.g., growth, fatness and reproductive performance. To limit the effect of enviromental influences on gene expression pattern, both pigs were housed and fed with the same conditions and were prepubescent at slaughter time. Furthermore we compared the results obtained with RNA-seq with microarray data published in a previous study [18]. Finally, we also identified polymorphic sites and genes that potentially showed allele specific expression.

## Results and Discussion

### Mapping

We obtained about 60 M of 50 bp paired-end reads from one lane of an Illumina GAIIx machine, about 30 M was derived from each sample (Data are archived at NCBI Sequence Read Archive (SRA) under Accession SRP008516). After ambiguous mapping (allowing for multi-hits) with Tophat [17] a total of 20 M reads for each sample were mapped against the reference pig genome (assembly 9), although only 10 M were classified as proper pairs. The rest (4 M) fell into either one of these categories: reads without a mapped mate pair, mate is mapped on the same strand or mates overlap. The most likely explanations of the large amount of improperly mapped reads are the poor quality of the current pig genome assembly and the stringency of the version of Tophat used here, as this version does not allow gaps for the mapping. In addition, any situation where the distance between the mates is larger than the confidence interval of the insert size distribution, could be interpreted as trans-splicing events [19], structural variants or simply mapping artifacts [20]. The total number fragments mapped with unambiguous mapping (1 hit per read) were 14 M for each sample; out of these, 7 M were classified as proper pairs. A comparison between ambiguous versus unambiguous mapping results obtained with Tophat is shown in Additional file 1.

### Annotation of reads and transcripts assembly

To calculate the proportion of reads mapping to annotated exons, we run S-MART (see methods). Surprisingly, with a minimum overlapping of 1 nucleotide, less than half of the reads (44.1%) mapped to annotated exons; a figure that drops even further (32.9%) when considering a minimum overlapping of 50 bp (the total read length). The rest of reads mapped to annotated introns (18.7%), or either 1 kb 5'upstream or 3'downstream of the annotated gene (26.6%) (Table 1). The poor quality of the annotation of the pig genome probably explains why a majority of the mapped reads (55.9%) do not overlap with any known exons.

**Table 1:** Summary of reads' annotation

	Large White	Iberian
**Exons**	9238572	9141162

**Introns**	3833320	3943866

**5' Upstream or 3'Downstream**	5383546	5708057

Number of reads with at least one overlapping nucleotide mapping to either exons, introns or within 1 kb of gene boundaries.

Moreover, after assembling the short reads into transcripts by Cufflinks [21], only 1.2% of them matched exactly with annotated exons. The remaining reads were classified as intergenic transcripts (36.1%), intron retention events (35.6%), contained in known isoforms (12.5%), pre-mRNA molecules (6.2%), polymerase run-on fragments (3.6%), putative novel isoforms of known genes (2.9%) and others (Table 2). These results unfortunately underline the incompleteness of the current annotation of the pig transcriptome and of its complexity.

**Table 2:** Transcripts assembly

	=	C	e	i	j	o	p	u	Total
**Large White**	2178	22243	11328	67989	5557	3866	6775	72288	192224

**(%)**	1.13	11.57	5.89	35.37	2.89	2.01	3.52	37.61	100

**Iberian**	2000	21580	10349	57623	4530	3341	5752	55617	160792

**(%)**	1.24	13.42	6.44	35.84	2.82	2.08	3.58	34.59	100

The number of transcripts assembled with Cufflinks and the percentage they represent in each sample. The high number of assembled transcripts is probably an artifact due to truncated Cufflinks assemblies. Class codes described by Cuffcompare: "=" Exactly equal to the reference annotation, "c " Contained in the reference, annotation, "e" possible pre-mRNA molecule, "i " An exon falling into a intron of the reference, "j " New isoforms, "o" Unknown, generic overlap with reference, "p" Possible polymerase run-on fragment, "u" Unknown, intergenic transcript.

### Annotating orthologs

A total of 4,124 novel transcripts (the real number of transcript units may be smaller, as less abundant transcripts receive less complete sequencing coverage resulting in numerous transfrags) were identified in intergenic regions (see methods). To investigate which of these transcripts actually encode a protein, we used Augustus [22] and found 714 novel putative proteins. We identified homologous DNA sequences (see methods) in *Bos taurus *and *Homo sapiens *genomes for most (413) of these novel proteins: 362 were orthologs with both cow and human, 20 with human only and 31 with the cow genome only. This result is consistent with *Bos taurus *being closer to *Sus scrofa *than human [23]. Interestingly, when we looked for homologous DNA regions within the *Sus scrofa *genome, 53 paralogous regions were detected (51 duplications and 2 present in three copies).

To find out whether the predicted proteins from the homologous regions were already annotated, we ran BLASTP against the *Homo sapiens*, *Bos taurus *and *Sus scrofa *protein databases (http://www.ensembl.org/info/data/ftp/index.html). Overall, we identified 38 novel computationally predicted and 344 known proteins for the human, 15 novel predicted and 378 known proteins for the cow and 653 novel predicted and 89 known proteins for the pig. The novel computationally predicted proteins found in the pig are now experimentally confirmed by RNA-seq. See Additional file 2 for the coordinates of orthologous and paralogous genes.

### Transposable elements

As many previous studies reported high activity of transposable elements (TE) in germlines [24-27], we ran RepeatMasker to identify repetitive elements in the pig genome and in the transcriptome of the testicles. The fraction of transposable elements expressed in male gonads (SINEs, LINEs, LTR and DNA elements), compared to the total number detected in the pig genome, is less than 3%. However, approximately 20% of the expressed transcripts units harbor at least 1 transposable element (8% of the bp sequenced). The type of TE being more active in both breeds, in terms of number of elements expressed divided by the total number present in the genome, is DNA transposons, but accounting just for the number of elements expressed is SINE family for Large White and the LINE family for Iberian. LINE1 elements also have been reported to contribute to the transcriptome in human somatic cells [28]. It is interesting to mention that 16% of protein-coding transcripts contain transposable elements in their sequence and they are transcribed in the same transcript unit. Apart from these interspersed repeats, hundreds of small RNA (tRNA, snRNA and rRNA) and thousands of simple and low complexity repeats were also identified in the transcriptome. The presence of non-polyadenylated RNA could be a remaining contamination as they are highly expressed molecules and difficult to remove completely. Another possible explanation is the presence of small functional RNAs embedded in the introns of polyadenylated molecules of pre-mRNA [29,30]. Detailed results of the repetitive elements detection with Repeatmasker are shown in Additional file 3a and 3b.

### LncRNA annotation

In order to define a set of putative lncRNAs in the pig transcriptome, we applied several filtering criteria. Using the procedure in [31] for the definition of lncRNA in humans, we excluded all transcripts mapping within 1 kb of an annotated protein-coding gene in the pig genome. This makes it less likely to consider the 5' or the 3' UTR of a protein-coding gene as a non-coding RNA. Yet, this filtering may not be stringent enough when dealing with insufficiently annotated genomes. For that reason, we further refined our analysis by excluding all transcripts coding for a complete proteins (according to Augustus). A third filter was applied by removing all transcripts having a hit against NR (BlastX), against Pfam (RPS-Blast) or against Rfam [32] (web-site batch search). The final filter was applied mapping all the resulting transcripts onto the human genome (the best annotated mammalian genome), and removing any transcript strongly overlapping with a protein-coding gene. The result is a dataset made of 2047 transcripts and referred in the rest of this text as the lncRNAs.

**Figure 1:**
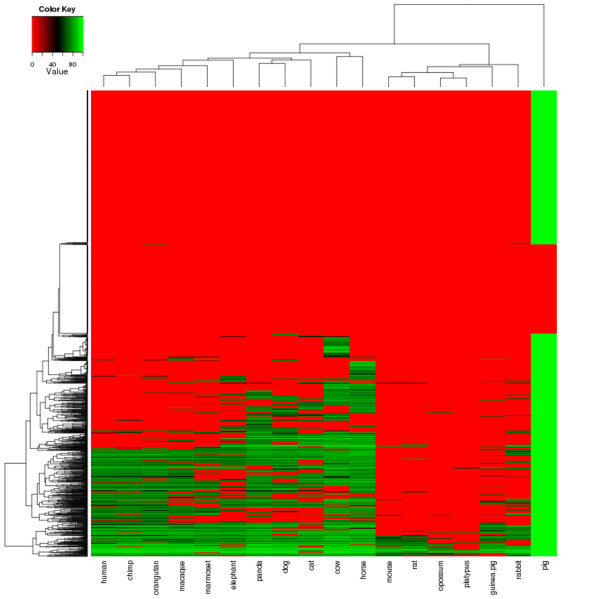
**LncRNAs mammal conservation**. The heatmap recapitulates the screening result of the new discovered 2047 pig lncRNAs versus eighteen mammal genomes. The columns represent the mammal genomes while the rows indicate the query lncRNAs. The spots indicate the result of the search of each pig lncRNA versus the different genomes. Green spots represent hits having high similarity scores. Black spots indicate low similarity scores. Red spots indicate that no homolog was detected.

The main problem when dealing with ncRNAs is to distinguish between spurious transcripts resulting from promoter leakiness and biologically functional transcripts. In order to do so, we assessed the level of evolutionary conservation of each lncRNA across the 18 available mammalian genomes. As shown in previous work, this conservation cannot be directly inferred from reference multiple genome alignment [31]. We therefore used a standard gene discovery strategy that relies on a combination of BlastN (version 2.0 MP, Gish, unpublished) and exonerate [33]. BlastN allows a rough identification of the location of each transcript in the considered genome while exonerate is used to precisely delineate the corresponding gene structure. We only considered as potential homologues hits for which exonerate alignments yield more than 70% coverage with the pig transcript. The results of this extensive homology based analysis are displayed on a heatmap (Figure 1).

**Figure 2:**
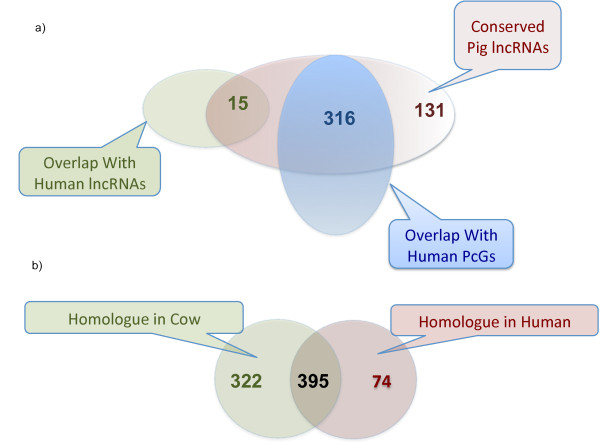
**Ven diagrams of the predicted homologues in human and cow**. **a) **469 pig lncRNA presented homology with human. 15 pig lncRNA overlap with human lncRNA, 316 overlap with human PcGs annotations and 131 lncRNA presented homology with unannotated human DNA regions. **b) **Comparison of lncRNAs having a homolog in human and in cow.

In the context of this analysis, we managed to map 986 transcripts in at least one other mammal species. A sizeable number of transcripts (391) were excluded because they contain pig repeats (red block in the pig column on Figure 1). The rest of the transcripts roughly fall in three categories. The first one is made up of genes apparently conserved across most tested mammals, including human. These make up a group of 469 genes (Figure 2). In this group, 131 transcripts map onto human genomic regions with no annotation. The rest either overlap with protein-coding genes (316), with known lncRNAs (15) or pseudogenes (7). It is important to note that an overlap with a PcG is not incompatible with a transcript being a lncRNA. The second category is made up of a group of 322 transcripts conserved among Artiodactyla (pig and cow) but not found in human. The last group encompasses all the putative lncRNAs for which no homologue was found in other species. While these may be pig specific, further analysis would be needed to confirm their biological relevance (for instance by testing their differential expression across tissues).

It is worth mentioning that the transcripts thus identified have a gene structure significantly different from their human counterparts. 97% are single exon genes and 2.5% bi-exonic, a figure significantly different from human where a much higher portion is bi-exonic. This finding may simply reflect insufficient coverage in the RNA-seq experiment resulting in truncated cufflinks models and thus should not be taken, so far, as strong evidence of distinct lncRNA organization between species.

It is in agreement with our observation that the lncRNA we observe in pig are roughly half the size of those reported in human (456 vs 925). As a consequence, the number of independent transcripts reported here is quite likely to be an over estimation.

### Gene expression analysis

In total, 12,816 annotated genes were expressed in gonads. Less than 1% of these genes were expressed more than 10000 FPKM; around 5% were expressed between 1000 -10000 FPKM, 50% between 10-1000 FPKM, 40% between 10-100 FPKM and 3% between 1-10 FPKM (Additional file 4). The rest were expressed below 1 FPKM. The maximum expression level of an annotated gene was 61,000 and 73,000 FPKM in Large White and Iberian, respectively. The gene ontologies of the 100 most expressed genes (mainly ribosomal proteins and heat-shock proteins) in both samples were related with transcription and translation, protein folding, lipid and cholesterol metabolism (apoproteins), induction of apoptosis and response to stress. These results are consistent with those observed in other mammalian species with RNA-seq [34].

The correlation of gene expression levels between both samples (Large White vs. Iberian) was very high (r = 0.85), which suggests that a large fraction of the transcriptome is conserved across individuals. This is consistent with our previous results which showed that the largest source of variability was tissue rather than sex or breed [18].

**Figure 3:**
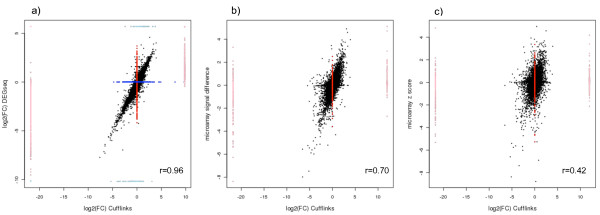
**Measuring gene expression**. a) DEGseq vs. Cufflinks estimates of log2 fold changes between Large White and Iberian expressed genes. Blue and red points correspond not expressed genes in microarrays and Cufflinks, respectively. Light blue and light red points correspond to microarray and Cufflinks infinite values. b) Microrray vs. RNA-Seq individual measurements. The microarray data correspond to signal intensity difference between Large White and Iberian, whereas the RNA-Seq measurement is the log2 fold change as obtained from Cufflinks. c) Microarray breed z-score values vs. RNA-Seq log2 fold change. The Pearson's correlations (r) were significant in each case (Pv < 2.2 × 10^-16^) and calculated considering only expressed genes and no infinite values.

Gene expression was quantified using two different approaches: DEGseq [35], which uses raw fragment counts per gene as a measure of expression, and Cufflinks [21], that uses an estimation of fragments per kilobase of exon per million reads mapped (FPKM). DEGseq's protocol recommends working only with the uniquely mapped fragments, whereas Cufflinks can deal with multiple mappable fragments. In this study, the correlation of the log2 of the fold change between both methods was 0.96 when discarding infinite values and taking expressed genes in both methods into account (see Figure 3a). Nevertheless, fragments mapping to homologous genes, which constitute 15%-20% of the expressed genes, are lost when considering fragments that map only once in the transcriptome, so it is arguable how to actually compare expression levels measured with these two programs.

We also compared the RNA-seq expression results with Affymetrix microarray data obtained in a previous study [18]. As many microarray probes may map to the same gene, the average probe value per gene was calculated. A total of 9,112 Ensembl ID genes could be retrieved from microarray probes data for RNA-seq comparisons. The correlation between the individual microarray signal intensity difference and the log2 of the fold change from RNAseq was quite high (r = 0.71, see Figure 3b). From the microarray study, we also had a Bayesian standardized breed score (z-score) available for each gene. When comparing the microarray breed z-score and the log2 of the fold change in RNA-seq, the correlation was also moderately high (Pearson correlation r = 0.46, see Figure 3c).

### Differential expression analysis

**Figure 4:**
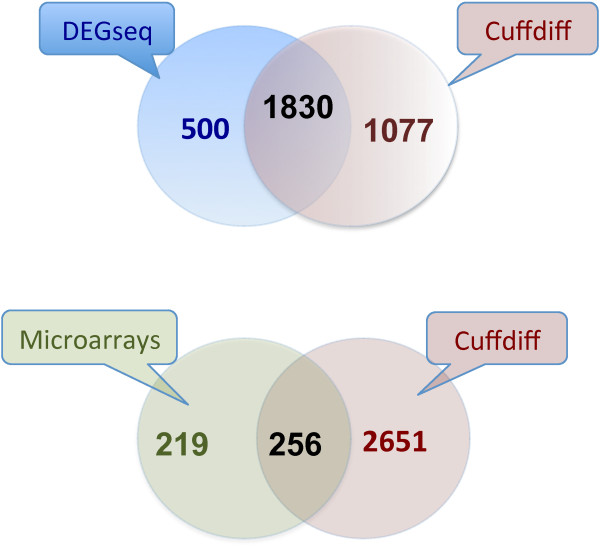
**Overlapping of differentially expressed genes**. Top: Differentially expressed genes identified by DEGseq and Cufflinks. Bottom: Differentially expressed genes identified by microarrays (breed z-scores) and RNA-Seq (Cufflinks).

We compared the performance of Cufflinks and DEGseq to detect differential expression between both samples (P < 0.001 and fold change > 2). Cufflinks identified 2,907 differentially expressed genes with multiple mappable fragments and DEGseq 2,330 with uniquely mapped fragments; there was a reasonable agreement between softwares, 1,830 genes (Figure 4, top). But, to be more conservative, and to try to get only differential expression due to breeds and not merely to stochastic reasons, we extracted differentially expressed genes from breed effects data, with absolute z score threshold > 1.65. Then we selected the intersection of RNA-Seq (Cufflinks) and microarrays reducing the number of differentially expressed genes to 256 (Figure 4, bottom). Out of these, 147 genes were over expressed in Large White and 109 in Iberian. Among differentially expressed genes, spermatogenesis, response to steroid hormone stimulus and sensory organ development were significantly over-represented children gene ontologies (P < 10^-3^). Doing the same analysis but considering the GOslim of the pig described in the methods section, we obtained an enrichment of reproduction, developmental process and fatty acid metabolic process parental gene ontologies (P < 10^-3^). Interestingly, among the significant KEGG-pathways represented, we found many differentially expressed genes in the PPAR signaling pathway, which is involved in lipid metabolism and, specifically, it has been shown to have a role in mice gonads fat deposition [36].

### Expression differences of coding and non-coding genes

**Figure 5:**
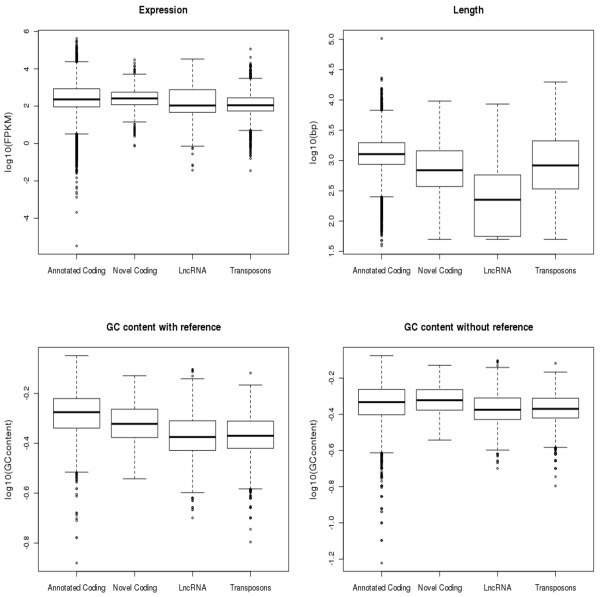
**Expression levels according to annotation**. a) Boxplots of expression level (log10 FKPM) for annotated coding genes, novel coding genes, lincRNA and transcripts with TE. The black line represents the median. b) Boxplots of the transcript unit length in base pairs (log10). c) Boxplots of the GC content (log10) using the reference annotation for transcriptome assembly. d) Boxplots of the GC content (log10) without using the reference annotation.

We also compared the expression level of the annotated coding genes, novel coding genes, lncRNA and transcripts containing at least one transposable element (see Figure 5a). The median expression level of annotated coding genes (230.1 FPKM) was slightly lower than of the novel-coding genes (258.0 FPKM). The range of expression levels of the annotated coding genes is, however, broader than that of the novel coding. We were able to detect annotated coding genes with very low expression levels, which highlights that fact that providing the reference gene models, it is easier to detect genes even at low coverage. Simultaneously, the expression median of transcripts units with at least one TE (111.6 FPKM) and lncRNAs (107.8 FPKM) is more than 50% lower than those of coding regions. As non-coding transcripts are probably involved in gene regulation, less number of copies is needed [37]. The annotated coding genes are on average longer than the novel coding (Figure 5b). This may be due to several reasons, first a higher coverage is needed to fully assemble a novel gene, but, it is has been also described than novel genes tend to be shorter than annotated ones. Overall we found that the average transcript length for protein-coding gene is 1578 bp, roughly half the size of transcripts in the human transcriptome (2982 bp). Interestingly, we observed a similar ratio when comparing the average size of lncRNAs in our experiment (456 bp) with that observed in human (925 bp). This fairly constant ratio suggests a homogenous bias, most likely the result of a lack of connecting paths between exons of the same transcript unit.

The GC content median of the coding genes (annotated 0.46 and novel 0.47) was the same but higher than the lncRNA (0.42) and transcripts harboring at least one TE (0.42) because coding genes tend to be rich in GC [38]. Important to notice is the fact that GC content of annotated genes differs depending on whether we provide to Cufflinks the reference gene annotations (Figure 5c) or not (Figure 5d). In the former, the GC content is much higher (0.53) than the latter (0.46), pointing to a possible bias towards AT during Illumina library preparation and sequencing workflow. Recently, a new amplification protocol has been published that solves this problem [39].

### SNP identification

We divided the SNPs in two classes, fixed, i.e. differences with respect to the assembly, a Duroc pig, and segregating when the individual was heterozygous. The number of SNPs found per bp sequenced is shown in Table 3. In autosomes, approximately the same amount of fixed SNP with respect to the Duroc genome reference is found in both breeds, but around 30% less divergence is found in Iberian on × chromosome. Regarding the segregating SNP, in autosomes, we found 30% less variability in Iberian than in Large White and almost 50% less variability in the × chromosome. This result is in agreement with the high inbreeding level of the Iberian strains. Fixed SNP and segregating SNP annotation is shown in Table 4 introns and 3' downstream regions of annotated genes were the most polymorphic, a result of less evolutive constrains than exonic and 5'upstream regions of the genome; 3'UTR was also more variable than 5'UTR regions. As expected, more SNP were synonymous than non-synonymous in CDS.

**Table 3:** SNP statistics

	Fixed (SNP/kb)	Segregating (SNP/kb)	Total (SNP/kb)
**Large White**	29558 0.64	11230 0.24	40788 0.88

**Iberian**	25668 0.59	7552 0.17	33220 0.76

Number of fixed SNP with respect to the reference genome and number of segregating SNP within each breed. Within brackets, the number of SNP per kb assembled.

**Table 4:** SNP annotation

	Fixed		Segregating	
	Large White	Iberian	Large White	Iberian

**Synonymous coding**	2083	1727	1352	757

**Non synonymous coding**	1073	910	852	494

**5' UTR**	150	77	73	39

**3' UTR**	1187	1029	1004	622

**Stop lost**	3	1	0	4

**Stop gained**	1	3	7	9

**Intronic**	15101	13020	3388	2515

**5'Upstream**	2983	2588	1176	628

**3'Downstream**	9440	8549	5579	3831

**Splice site**	311	259	91	86

**Non synonymous coding**	1073	910	852	494

**Within non coding gene**	262	268	14	9

**Intergenic**	5847	5042	1539	1076

The number of SNP is degenerated; each SNP can have more than one annotation.

### Allele specific expression

**Figure 6:**
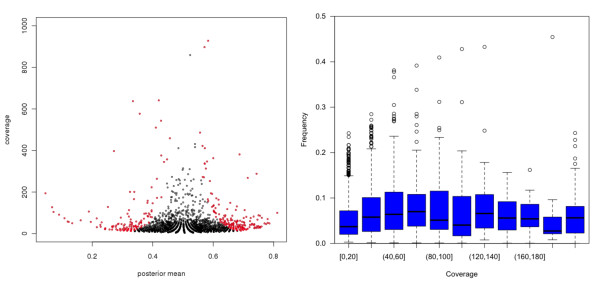
**Allele specific expression**. a) Coverage versus posterior mean of allele transcription rate (*p*); each point represents a SNP; red points are SNP showing significant ASE and black points are SNPs with no significant ASE. b) Barplot of coverage versus absolute value of *p*. It can be seen that there was not a consistent relation between ASE and coverage.

A beta binomial model was applied to detect allele specific expression ASE (see methods). A total of 428 SNP (3.8%) with average coverage of 55 × and 338 SNP (4.5%) with average coverage 121 × showed allelic imbalance in Large White and Iberian samples, respectively. Coordinates and annotation of SNPs with significant results are listed in Additional file 5. Figure 6 shows the relation between coverage and the posterior mean of allele specific expression *p *(see methods). Figure 6a indicates how, although very extreme values of *p *are always significant, intermediate values (*p *between 0.3 - 0.4 and 0.6 - 0.7 approximately) are significant only if enough coverage exists. This is a result of how the prior (*p *= 0.5) is dominated by empirical evidence as data increases. Figure 6b was plotted to show that an increased coverage does not result in an average higher ASE and therefore significance is not a statistical artifact. Further, we did not observe either any consistent higher expression of the reference vs. the alternative allele (results not shown) and therefore it is not an alignment artifact either.

Several SNP with significant ASE are located contiguously within intergenic regions, suggesting the presence of putative functional units not yet annotated in the pig genome. There were not many genes with ASE shared between the two samples, likely due to different genotypes at the regulatory motif of the two breeds. There were only 22 common SNPs exhibiting ASE in both animals, but in three of the SNPs we observed over expression of different alleles in each breed. Logically, these results should be taken as statistical evidence, genotyping or sequencing the cis-regulatory motives and linkage disequilibrium information are, however, needed to confirm whether these SNPs show genuine ASE.

## General Discussion

We present the first, to our knowledge, comprehensive exploration of the pig gonad transcriptome carried out with RNA-seq, a technology that offers critical advantages over microarray. Importantly, RNA-seq allows us to improve dramatically the annotation of the species and the discovery of new splicing events. Here, we have confirmed that a large part of the transcription effort in the cell is spent on TE sequences. A recent RNA-seq study in human and primate brain transcriptomes also found high proportion of reads mapping to repetitive elements, mainly from the Alu family [40]. Previous works in mice also indicated high expression of TE in germlines. The number of TE is probably an over-estimation as we did an ambiguous mapping reporting only the best alignments. On the other side, Cufflinks down weights the expression level taking into account mapping uncertainty [21].

Unfortunately, we also confirm that current porcine annotation is incomplete, as evidenced by read mapping annotation: more than 50% of the fragments do not map to annotated exons. The fact that many reads map to introns could be explained either by intron retention (new isoforms) or pre-mRNA presence. Reads mapping outside the boundaries of annotated genes could be explained either by polymerase run-on fragments or a bad annotation of the gene endings. Many intergenic reads have been mapped to putative novel coding transcripts, some of them presenting orthology with related species. The poor status of the annotation is confirmed by the presence of 104 highly conserved transcripts, that would have been annotated as lncRNAs if we had only considered the pig annotation, but whose homologues in human show a perfect overlap with protein-coding genes.

Given that we had previously analyzed the transcriptome of a wider collection of pig and tissues with Affymetrix microarrays [18], we were able to compare both technologies. The correlation within individuals was rather high (r = 0.71) and comparable to other reported studies [6,41-44]. Furthermore, the correlation of expression between Iberian and Large White obtained with microarray (employing all animals) and the individuals (obtained with RNAseq) was also moderately high (r = 0.46), suggesting that transcriptome patterns are relatively stable. Among the most differentially expressed genes, those involved in spermatogenesis and lipid metabolism are over-represented, which may be a result of targeted tissue selection. It is noteworthy that Large White and Iberian breeds are phenotypically extreme for both reproduction and fat deposition traits so these data would suggest a correlated effect on the regulation of genes involved in these traits.

In general, Cufflinks has a better performance to map fragments to genes or isoforms that are physically overlapping or very similar in sequence, as it uses a statistical model to deal with multiply mapping fragments. DEGseq works with uniquely mapped reads, thus underestimating gene expression levels of homologous genes but also discarding those reads belonging to two overlapping genes; a bias in expression level is thus introduced in these cases. The algorithm behind Cufflinks is rather naïve, though. Recently, new approaches that implement improved algorithms to deal with ambiguously mapped reads data and avoid bias in downstream analysis have been published [45,46].

Although not the main purpose of the work, we also found a lower rate of heterozygosity in Iberian than in the Large White animal, in agreement with the fact that Iberian pigs are normally inbred. Finally, we also explored ASE, a topic that has received a renewed interest recently. In this study, ~ 4% of the segregating SNP presented allelic imbalance. From these, around 40% were located inside annotated genes, the rest were located in blocks in intergenic regions, pointing to putative functional transcripts. To be able to confirm ASE, more animals should be tested because the majority of the SNPs with ASE were not common between Large White and Iberian pigs.

## Conclusions

We provide a complete survey of the pig male gonad transcriptome and identified many novel elements. However, to further improve the annotation of the pig genome, a large effort from the community will be necessary by sequencing more tissues at different developmental stages. In order to detect novel splicing events and to reconstruct novel isoforms, RNA-seq studies with very high coverage are required. Here, we also have shed some light on the dark matter of the transcriptome; in particular, we remark the discovery of novel long non-coding transcripts and the fact that TE expression seems to take a large fraction of the transcriptome. Their precise roles need to be elucidated in future studies. We also show that correlation between microarray and RNAseq expression data are reasonably high (linear correlation r = 0.71). Finally, Large White and Iberian pigs seem to have diverged most for genes involved in spermatogenesis and lipid metabolism, not only in terms of gene expression but also phenotypically. Interestingly, it is well known that genes related to gametogenesis are subject often to a positive selection rate [47,48]. More work is required to investigate whether the differences in expression in these genes are adaptive.

## Methods

### Animal material

Animal material is fully described in [49]. The two animals were housed and slaughtered simultaneously. Animals were prepubescent, three months of age, and weights were 45.0 and 30.1 kg for Large White and Iberian animals, respectively.

### Library preparation

Total RNA from gonads was extracted as described in [49]. Briefly, Total RNA was extracted from 100 mg tissue using the RiboPure™ kit (Ambion, Austin, USA). RNA integrity was assessed by Agilent Bioanalyser 2100 and RNA Nano 6000 Labchip kit (Agilent Technologies, Palo Alto, USA). Due to high variation in concentrations of the total RNA obtained in different tissues, all samples were concentrated and cleaned using the RNAeasy MiniElute Cleanup kit (Qiagen, Basel, Switzerland) obtaining final concentrations between 500 and 1000 ng/μl. Sequencing libraries were produced using the Illumina mRNA-Seq sample preparation kit, following the manufacturer's instructions. Briefly, 4 μg of total RNA were used as input for poly-A+ selection, followed by metal-catalyzed fragmentation of the selected mRNA (peak of size distribution at approx. 240 nt). After reverse transcription to cDNA using random hexamer primers, we performed end-repair and A-tailing of the double stranded cDNA. Large White and Iberian cDNA were ligated to indexed pairs of adapters, see Additional file 6. The cDNA was size selected on a 2% agarose gel, and fragments corresponding to an insert size of 237 nucleotides were excised from the gel. The DNA was recovered from the gel slice using QIAquick gel extraction kit (Qiagen). Therafter, the libraries were amplified in 15 cycles of PCR using primers Illumina 1.0 and Illumina 2.0. The libraries were quantified using Taqman, and pooled at a concentration of 10 pM. We performed paired-end sequencing of the libraries on the Genome Analyzer IIx using Illumina v4 sequencing chemistry.

### Reads annotation

S-MART (http://urgi.versailles.inra.fr/Tools/S-MART) was used to count the number of reads mapping to exons, introns and 1 kb upstream/downstream of the annotated genes. A minimum overlapping of 1 nucleotide was chosen to declare an overlap.

### Mapping, Assembling and Quantifying

Reads were mapped against the pig reference genome (assembly9) with Tophat v.1.0.14 [17] using the following settings: maximum of 40 hits per read (reporting best alignments), expected mean inner distance between mate pairs of 137 and a standard deviation for the distribution on inner distances between mate pairs of 100. For unambiguous mapping of the reads, the maximum alignments per read were set to 1. Sequence statistics were analyzed with FASTQC (http://www.bioinformatics.bbsrc.ac.uk/projects/fastqc). Base sequence qualities and proportion of bases per cycle are shown in Additional files 7a and 7b. A decrease in base quality is observed towards the end of the sequence and there is a bias in nucleotide content in the first 10 cycles of the reads due to the random hexamer primer library preparation approach [50]. Recently, a new statistical approach has been proposed to solve this bias [51]. Transcripts were assembled and quantified by Cufflinks v.0.9.0 [21]. To improve the robustness of the differential expression estimates the quartile normalization was used and the contribution of the top 25 percent most highly expressed genes was excluded (-N option). The minimum alignment count per locus was set to 20 (-c option).

### Orthology detection

Intergenic expressed regions not yet annotated in the pig genome were extracted with Cuffcompare [21] and custom Python and R scripts. Only those regions expressed in both samples were considered for a conservative approach. To identify putative coding transcripts, we run Augustus [22] providing exon boundaries and allowing only complete proteins translations from the forward strand.

### Transposable element analysis

We run RepeatMasker (http://www.repeatmasker.org/) with options 'quick search' and species 'pig' to identify repetitive and transposable elements (TE) in pig genome and male gonads transcriptome. We used RepeatMasker version open-3.2.9, rmblastn version (1.2) 2.2.23 and RepBase update 20090604.

### LncRNA identification

All the transcripts not overlapping with pig protein-coding genes and falling at least 1 kb away from the closest protein annotation were considered for our analysis. A series of filtering steps were then implemented. The first one consisted in selecting the transcripts for which Augustus returned no (or just partial) coding potential. BlastX (NCBI Package version 2.2.25) was then used to search all possible translational products (the six possible reading frames) of each transcript against the NCBI non-redundant protein database (last update 05/29/2011). All the transcript queries that matched a known protein with an expectation value lower than 10^-5 ^were discarded. Likewise, RPS-Blast (NCBI Package version 2.2.25) was used to search the possible translational products of each transcript against a database of Pfam profiles [52] and the transcripts returning an expectation value lower than 10^-5 ^were removed. In order to filter the transcripts belonging to known classes of RNAs (snoRNAs, tRNAs, etc...), all the sequences were sought against Rfam (Release 10.0) using the Rfam searching facility available at: http://rfam.sanger.ac.uk/search#tabview=tab0.

Finally the remaining transcripts were remapped against the human genome and the homologous positions were intersected with protein-coding gene annotations (GENCODE version 3c). The screening was performed using a combination of BlastN and exonerate (as described in the screening pipeline in the methods). The transcripts whose human homologue resulted to be fully included in protein-coding exons were removed.

### Screening pipeline

The screening pipeline was composed by three phases. The first consisted in seeking each query against the target genomes with a version of BlastN optimized for ncRNAs discovery [53]. Secondly, using exonerate each query was realigned versus the genomic regions pointed by Blast. For each query and for each genome was kept just the best hit that was successfully realigned. The exonerate alignments spanning for at least the 70% of the pig queries were retained. Finally, each query was compared versus all the putative discovered homologs by realigning the transcripts sequences with T-Coffee [54] and measuring the query/homolog pairwise similarity.

### Differential expression (DE) analysis

To test DE with unambiguous mapping data DEGseq was used [35]. MA plot-based method (where M is the log ratio of the counts between two experimental conditions for gene g, and A is the two group average of the log concentrations of the gene) with a random sampling method (MARS) was selected. To count the number of fragments that uniquely map to an exon, HTseq-count was used with 'union' as overlapping mode, 'gene' as feature and not strand-specific. A locus was considered as expressed if it had a minimum count of 40 fragments (summing the reads in both samples). From a total of 9 M unambiguously mapped reads for each library, 4.5 M of reads felt in the category of 'no_feature' (no annotation provided). The software discarded reads mapping to two overlapping genes (20,000 reads). Cuffdiff [21] was used to test DE using same options as discussed above for ambiguous mapping data.

In the microarray assay, we employed the GCRMA normalization method [49] and a Bayesian z-score measure as detailed in [55]. Briefly, normalized data were analyzed with model

y=Tissue+Breed+Sex+Probeset+PT+PB+PS+Residual,

where *PT*, *PB *and *PS *stand for the probeset × tissue, probeset × breed and probeset × sex interactions, respectively. The Bayesian breed z-score for the g-th probeset is defined as *z_g _*= E(*PB*_g_|y)/SD(*PB*_g_|y), where E(*PB*_gj_|y) and SD(*PB*_gj_|y) are the expected and SD values of the posterior distribution of PB, respectively [55].

### Gene ontology analysis

Parental gene ontology enrichment analysis was performed with the QuickGO browser (http://www.ebi.ac.uk/QuickGO/) using a GOSlim extracted from the AmiGO browser (http://amigo.geneontology.org/cgi-bin/amigo/go.cgi) and made up of 23 parental pig GO: biological regulation, cellular process, metabolic process, multicellular organismal process, developmental process, signaling, localization, response to stimulus, immune system process, cellular component organization, reproduction, biological adhesion, cellular component biogenesis, death, locomotion, multi-organism process, growth, pigmentation, rhythmic process, viral reproduction and cell killing. Expected and observed GO percentages were compared with a Fisher's exact test as implemented in R. To test for an enrichment of specific ontology categories, we simply computed a two-sided *t*-test assuming a normal distribution for number of counts. The children gene ontology enrichment and KEGG pathway analyses were performed with the DAVID database (http://david.abcc.ncifcrf.gov/). Prior to GO analysis, the pig gene IDs were converted to human gene IDs with Biomart (http://www.biomart.org/) as the database had poor pig Ensembl annotations. The list of differentially expressed genes (intersection of Cufflinks and microarray breed effects) was compared against total expressed genes in male gonads (background).

### SNP identification

**S**NPs were identified from unambiguously mapped reads using Samtools (http://samtools.sourceforge.net/). The minimum SNP quality was 10 and the minimum read depth was set to 3 × for fixed SNP with respect to the reference and 4 × for segregating SNP. As many false SNP were located at the splice sites due to the difficulties of alignments near indels (splicing sites), they were removed from the final set. Annotation of the SNP was made with custom Perl scripts using the Ensembl APIs.

### Allele specific expression

To test for allele specific expression heterozygous SNP were selected from both samples using uniquely mapped reads (SNP quality > 10, minimum depth of 4x, minimum allele count of 2). Allele specific expression can be inferred when, in a heterozygous site, one allele is transcribed at significantly higher or lower rate (*p*) than the other allele. We used a beta - binomial model within a Bayesian framework to infer whether *p *was significantly different from 0.5. The posterior probability of *p *is given by the distribution

$$\text{Be}(\alpha +n_{a},\;\beta +n-n_{a})\;\text{B}\left(n_{a},n\right)\times \text{Be}(\alpha ,\beta ),$$

where Be() is a beta distribution; B(), a binomial; *n *is the number of reads for that SNP; *n_a _*, the number of reads pertaining to one arbitrary allele, and *α *and *β *are hyperparameters. The data was fitted using an empirical Bayesian approach such that the mean and variance of Be(*α*, *β*) were those observed in the real data. The obtained *α *and *β *were 4.99 and 3.84 in Large White and 6.38 and 6.20 in the Iberian data, respectively. ASE was considered when the 95% Highest Density Region (HDR) did not include *p *= 0.5. HDR was computed with function "HDIofICDF.R" in R (http://www.indiana.edu/~kruschke/DoingBayesianDataAnalysis/Programs/HDIofICDF.R).

## Abbreviations

TE: Transposable elements, LncRNA: Long non-coding RNA, DE: Differential expression, UTR: Untranslated region, FPKM: Fragments Per Kilobase of exon model per Million mapped fragments, CDS: Coding sequence, ASE: Allele specific expression, GO: Gene ontology, PPAR: Peroxisome proliferator-activated receptor, KEGG: Kyoto Encyclopedia of genes and genomes, SNP: Single nucleotide polymorphism, APIs: Application programming interface, PcGs: Protein-coding genes.

## Authors' contributions

MPE conceived and supervised research and provided material. AEC, RK, NP, GB and CN analyzed data. All authors discussed the results and wrote and approved the final manuscript.

## Supplementary Material

Additional file 1 **Mapping statistics**. Comparison between ambiguous and unambiguous mapping. Click here for file

Additional file 2 **Orthologs coordinates**. Sheet 1: Coordinates of putative novel coding genes in Sus scrofa transcriptome; sheet 2: *Bos taurus *orthologs; sheet 3: *Homo sapiens *orthologs; sheet 4: *Sus scrofa *paralogs. Click here for file

Additional file 3 **RepeatMasker results**. RepeatMasker results. A) Transcriptome analysis. B) Genome analysis. Click here for file

Additional file 4 **Expression range**. Abundance of annotated genes expressed between 1-10 FPKM, 10-100 FPKM, 100-1000 FPKM, 1000-10000 FPKM or more than 10000 FPKM. Click here for file

Additional file 5 **Annotation of allelic specific expression**. Coordinates and annotation of SNP with significant ASE results. Sheet 1: Large White results; sheet 2: Iberian results; sheet 3: Shared SNPs with ASE. Click here for file

Additional file 6 **Sequence of the adapters**. Where "P" refers to a PO4 moiety and * indicates a phosphorothioate bond. Click here for file

Additional file 7 **Quality control of the reads**. A) Raw reads quality control: Base qualities per cycle. B) Library sequencing bias: Proportion of bases incorporated in each sequencing cycle. Click here for file

## References

[B1] Jacquier A The complex eukaryotic transcriptome: unexpected pervasive transcription and novel small RNAs Nat Rev Genet 2009 10 12 833 844 19920851 10.1038/nrg2683

[B2] Shabalina SA Spiridonov NA The mammalian transcriptome and the function of non-coding DNA sequences Genome Biol 2004 5 4 105 10.1186/gb-2004-5-4-105 15059247 PMC395773

[B3] Lindberg J Lundeberg J The plasticity of the mammalian transcriptome Genomics 95 1 1 6 10.1016/j.ygeno.2009.08.010 19716875

[B4] Gustincich S Sandelin A Plessy C Katayama S Simone R Lazarevic D Hayashizaki Y Carninci P The complexity of the mammalian transcriptome J Physiol 2006 575 Pt 2 321 332 16857706 10.1113/jphysiol.2006.115568 PMC1819450

[B5] Guttman M Garber M Levin JZ Donaghey J Robinson J Adiconis X Fan L Koziol MJ Gnirke A Nusbaum C Ab initio reconstruction of cell type-specific transcriptomes in mouse reveals the conserved multi-exonic structure of lincRNAs Nat Biotechnol 28 5 503 510 10.1038/nbt.1633 PMC2868100 20436462

[B6] Mortazavi A Williams BA McCue K Schaeffer L Wold B Mapping and quantifying mammalian transcriptomes by RNA-Seq Nat Methods 2008 5 7 621 628 10.1038/nmeth.1226 18516045 PMC13303166

[B7] Toung JM Morley M Li M Cheung VG RNA-sequence analysis of human B-cells Genome Res10.1101/gr.116335.110 PMC3106332 21536721

[B8] Wang ET Sandberg R Luo S Khrebtukova I Zhang L Mayr C Kingsmore SF Schroth GP Burge CB Alternative isoform regulation in human tissue transcriptomes Nature 2008 456 7221 470 476 10.1038/nature07509 18978772 PMC2593745

[B9] Nicolae M Mangul S Mandoiu II Zelikovsky A Estimation of alternative splicing isoform frequencies from RNA-Seq data Algorithms Mol Biol 6 1 9 10.1186/1748-7188-6-9 PMC3107792 21504602

[B10] Wen J Chiba A Cai X Computational identification of tissue-specific alternative splicing elements in mouse genes from RNA-Seq Nucleic Acids Res 38 22 7895 7907 10.1093/nar/gkq679 PMC3001057 20685814

[B11] Gan Q Chepelev I Wei G Tarayrah L Cui K Zhao K Chen X Dynamic regulation of alternative splicing and chromatin structure in Drosophila gonads revealed by RNA-seq Cell Res 20 7 763 783 10.1038/cr.2010.64 PMC2919574 20440302

[B12] Wang L Xi Y Yu J Dong L Yen L Li W A statistical method for the detection of alternative splicing using RNA-seq PLoS One 5 1 e8529 10.1371/journal.pone.0008529 PMC2798953 20072613

[B13] Bottomly D Walter NA Hunter JE Darakjian P Kawane S Buck KJ Searles RP Mooney M McWeeney SK Hitzemann R Evaluating Gene Expression in C57BL/6J and DBA/2J Mouse Striatum Using RNA-Seq and Microarrays PLoS One 6 3 e17820 10.1371/journal.pone.0017820 PMC3063777 21455293

[B14] McManus CJ Coolon JD Duff MO Eipper-Mains J Graveley BR Wittkopp PJ Regulatory divergence in Drosophila revealed by mRNA-seq Genome Res 20 6 816 825 10.1101/gr.102491.109 PMC2877578 20354124

[B15] Graveley BR Brooks AN Carlson JW Duff MO Landolin JM Yang L Artieri CG van Baren MJ Boley N Booth BW The developmental transcriptome of Drosophila melanogaster Nature 471 7339 473 479 10.1038/nature09715 PMC3075879 21179090

[B16] Daines B Wang H Wang L Li Y Han Y Emmert D Gelbart W Wang X Li W Gibbs R The Drosophila melanogaster transcriptome by paired-end RNA sequencing Genome Res 21 2 315 324 10.1101/gr.107854.110 PMC3032934 21177959

[B17] Trapnell C Pachter L Salzberg SL TopHat: discovering splice junctions with RNA-Seq Bioinformatics 2009 25 9 1105 1111 10.1093/bioinformatics/btp120 19289445 PMC2672628

[B18] Ferraz AL Ojeda A Lopez-Bejar M Fernandes LT Castello A Folch JM Perez-Enciso M Transcriptome architecture across tissues in the pig BMC Genomics 2008 9 173 10.1186/1471-2164-9-173 18416811 PMC2335121

[B19] Herai RH Yamagishi ME Detection of human interchromosomal trans-splicing in sequence databanks Brief Bioinform 11 2 198 209 10.1093/bib/bbp041 19955235

[B20] McManus CJ Duff MO Eipper-Mains J Graveley BR Global analysis of trans-splicing in Drosophila Proc Natl Acad Sci USA 107 29 12975 12979 10.1073/pnas.1007586107 PMC2919919 20615941

[B21] Trapnell C Williams BA Pertea G Mortazavi A Kwan G van Baren MJ Salzberg SL Wold BJ Pachter L Transcript assembly and quantification by RNA-Seq reveals unannotated transcripts and isoform switching during cell differentiation Nat Biotechnol 28 5 511 515 10.1038/nbt.1621 PMC3146043 20436464

[B22] Stanke M Diekhans M Baertsch R Haussler D Using native and syntenically mapped cDNA alignments to improve de novo gene finding Bioinformatics 2008 24 5 637 644 10.1093/bioinformatics/btn013 18218656

[B23] Cooper GM Brudno M Green ED Batzoglou S Sidow A Quantitative estimates of sequence divergence for comparative analyses of mammalian genomes Genome Res 2003 13 5 813 820 10.1101/gr.1064503 12727901 PMC430923

[B24] Shi X Seluanov A Gorbunova V Cell divisions are required for L1 retrotransposition Mol Cell Biol 2007 27 4 1264 1270 10.1128/MCB.01888-06 17145770 PMC1800731

[B25] Zamudio N Bourc'his D Transposable elements in the mammalian germline: a comfortable niche or a deadly trap? Heredity 105 1 92 104 10.1038/hdy.2010.53 20442734

[B26] Branciforte D Martin SL Developmental and cell type specificity of LINE-1 expression in mouse testis: implications for transposition Mol Cell Biol 1994 14 4 2584 2592 8139560 10.1128/mcb.14.4.2584-2592.1994 PMC358626

[B27] Garcia-Perez JL Marchetto MC Muotri AR Coufal NG Gage FH O'Shea KS Moran JV LINE-1 retrotransposition in human embryonic stem cells Hum Mol Genet 2007 16 13 1569 1577 10.1093/hmg/ddm105 17468180

[B28] Rangwala SH Zhang L Kazazian HH Jr Many LINE1 elements contribute to the transcriptome of human somatic cells Genome Biol 2009 10 9 R100 10.1186/gb-2009-10-9-r100 19772661 PMC2768975

[B29] Donath A Findeiβ S Hertel J Marz M Otto W Schulz C Stadler PF Wirth S Caetano-Anollés G Noncoding RNA Evolutionary Genomics and Systems Biology 2010 Hoboken, NJ, USA: John Wiley & Sons;

[B30] Zamboni M Scarabino D Tocchini-Valentini GP Splicing of mRNA mediated by tRNA sequences in mouse cells RNA 2009 15 12 2122 2128 10.1261/rna.1841609 19850909 PMC2779668

[B31] Orom UA Derrien T Beringer M Gumireddy K Gardini A Bussotti G Lai F Zytnicki M Notredame C Huang Q Long noncoding RNAs with enhancer-like function in human cells Cell 143 1 46 58 10.1016/j.cell.2010.09.001 PMC4108080 20887892

[B32] Gardner PP Daub J Tate JG Nawrocki EP Kolbe DL Lindgreen S Wilkinson AC Finn RD Griffiths-Jones S Eddy SR Rfam: updates to the RNA families database Nucleic Acids Res 2009 37 Database issue D136 140 10.1093/nar/gkn766 PMC2686503 18953034

[B33] Slater GS Birney E Automated generation of heuristics for biological sequence comparison BMC Bioinformatics 2005 6 31 10.1186/1471-2105-6-31 15713233 PMC553969

[B34] Ramskold D Wang ET Burge CB Sandberg R An abundance of ubiquitously expressed genes revealed by tissue transcriptome sequence data PLoS Comput Biol 2009 5 12 e1000598 10.1371/journal.pcbi.1000598 20011106 PMC2781110

[B35] Wang L Feng Z Wang X Zhang X DEGseq: an R package for identifying differentially expressed genes from RNA-seq data Bioinformatics 26 1 136 138 10.1093/bioinformatics/btp612 19855105

[B36] Tsai YS Tsai PJ Jiang MJ Chou TY Pendse A Kim HS Maeda N Decreased PPAR gamma expression compromises perigonadal-specific fat deposition and insulin sensitivity Mol Endocrinol 2009 23 11 1787 1798 10.1210/me.2009-0073 19749155 PMC2775941

[B37] Orom UA Derrien T Guigo R Shiekhattar R Long Noncoding RNAs as Enhancers of Gene Expression Cold Spring Harb Symp Quant Biol10.1101/sqb.2010.75.058 PMC3779064 21502407

[B38] Arhondakis S Auletta F Torelli G D'Onofrio G Base composition and expression level of human genes Gene 2004 325 165 169 14697521 10.1016/j.gene.2003.10.009

[B39] Aird D Ross MG Chen WS Danielsson M Fennell T Russ C Jaffe DB Nusbaum C Gnirke A Analyzing and minimizing PCR amplification bias in Illumina sequencing libraries Genome Biol 12 2 R18 10.1186/gb-2011-12-2-r18 PMC3188800 21338519

[B40] Xu AG He L Li Z Xu Y Li M Fu X Yan Z Yuan Y Menzel C Li N Intergenic and repeat transcription in human, chimpanzee and macaque brains measured by RNA-Seq PLoS Comput Biol 6 e1000843 10.1371/journal.pcbi.1000843 PMC2895644 20617162

[B41] Fu X Fu N Guo S Yan Z Xu Y Hu H Menzel C Chen W Li Y Zeng R Estimating accuracy of RNA-Seq and microarrays with proteomics BMC Genomics 2009 10 161 10.1186/1471-2164-10-161 19371429 PMC2676304

[B42] Bradford JR Hey Y Yates T Li Y Pepper SD Miller CJ A comparison of massively parallel nucleotide sequencing with oligonucleotide microarrays for global transcription profiling BMC Genomics 11 282 10.1186/1471-2164-11-282 PMC2877694 20444259

[B43] Bloom JS Khan Z Kruglyak L Singh M Caudy AA Measuring differential gene expression by short read sequencing: quantitative comparison to 2-channel gene expression microarrays BMC Genomics 2009 10 221 10.1186/1471-2164-10-221 19435513 PMC2686739

[B44] Marioni JC Mason CE Mane SM Stephens M Gilad Y RNA-seq: an assessment of technical reproducibility and comparison with gene expression arrays Genome Res 2008 18 9 1509 1517 10.1101/gr.079558.108 18550803 PMC2527709

[B45] Ji Y Xu Y Zhang Q Tsui KW Yuan Y Norris C Jr Liang S Liang H BM-Map: Bayesian Mapping of Multireads for Next-Generation Sequencing Data Biometrics10.1111/j.1541-0420.2011.01605.x PMC3190637 21517792

[B46] Pasaniuc B Zaitlen N Halperin E Accurate estimation of expression levels of homologous genes in RNA-seq experiments J Comput Biol 18 3 459 468 10.1089/cmb.2010.0259 21385047

[B47] Haerty W Jagadeeshan S Kulathinal RJ Wong A Ravi Ram K Sirot LK Levesque L Artieri CG Wolfner MF Civetta A Evolution in the fast lane: rapidly evolving sex-related genes in Drosophila Genetics 2007 177 3 1321 1335 10.1534/genetics.107.078865 18039869 PMC2147986

[B48] Jagadeeshan S Singh RS Rapidly evolving genes of Drosophila: differing levels of selective pressure in testis, ovary, and head tissues between sibling species Mol Biol Evol 2005 22 9 1793 1801 10.1093/molbev/msi175 15917496

[B49] Perez-Enciso M Ferraz AL Ojeda A Lopez-Bejar M Impact of breed and sex on porcine endocrine transcriptome: a bayesian biometrical analysis BMC Genomics 2009 10 89 10.1186/1471-2164-10-89 19239697 PMC2656523

[B50] Hansen KD Brenner SE Dudoit S Biases in Illumina transcriptome sequencing caused by random hexamer priming Nucleic Acids Res 38 12 e131 10.1093/nar/gkq224 PMC2896536 20395217

[B51] Roberts A Trapnell C Donaghey J Rinn JL Pachter L Improving RNA-Seq expression estimates by correcting for fragment bias Genome Biol 12 3 R22 10.1186/gb-2011-12-3-r22 PMC3129672 21410973

[B52] Finn RD Mistry J Tate J Coggill P Heger A Pollington JE Gavin OL Gunasekaran P Ceric G Forslund K The Pfam protein families database Nucleic Acids Res 38 Database issue D211 222 10.1093/nar/gkp985 PMC2808889 19920124

[B53] Freyhult EK Bollback JP Gardner PP Exploring genomic dark matter: a critical assessment of the performance of homology search methods on noncoding RNA Genome Res 2007 17 1 117 125 17151342 10.1101/gr.5890907 PMC1716261

[B54] Notredame C Higgins DG Heringa J T-Coffee: A novel method for fast and accurate multiple sequence alignment J Mol Biol 2000 302 1 205 21 10.1006/jmbi.2000.4042 10964570

[B55] Irizarry RA Bolstad BM Collin F Cope LM Hobbs B Speed TP Summaries of Affymetrix GeneChip probe level data Nucleic Acids Res 2003 31 4 e15 10.1093/nar/gng015 12582260 PMC150247

