# Supplementary material for: Exploring the gonad transcriptome of two extreme male pigs with RNA-seq
Source: BMC Genomics. 2011 Nov 8;12:552. doi: 10.1186/1471-2164-12-552 (PMC3221674; doi:10.1186/1471-2164-12-552)
Supplement: Additional file 1 — Mapping statistics. Comparison between ambiguous and unambiguous mapping. [file 1471-2164-12-552-S1.PDF]

	Ambiguous mapping		Unambiguous mapping	
	Large White	Iberian	Large White	Iberian
**Proper pair**	11620776	10845574	8126448	7460544
**Improper pair**	9480342	9714594	6723675	6961220
Unmapped pair	4145310	4238558	3382287	3476522
Same strand	598176	664716	269072	332552
Overlapping	4736856	4811320	3072316	3152146
**Total mapped**	21101118	20560168	14850123	14421764
