# Supplementary figures and images for: Exploring the gonad transcriptome of two extreme male pigs with RNA-seq
Source: BMC Genomics. 2011 Nov 8;12:552. doi: 10.1186/1471-2164-12-552 (PMC3221674; doi:10.1186/1471-2164-12-552)

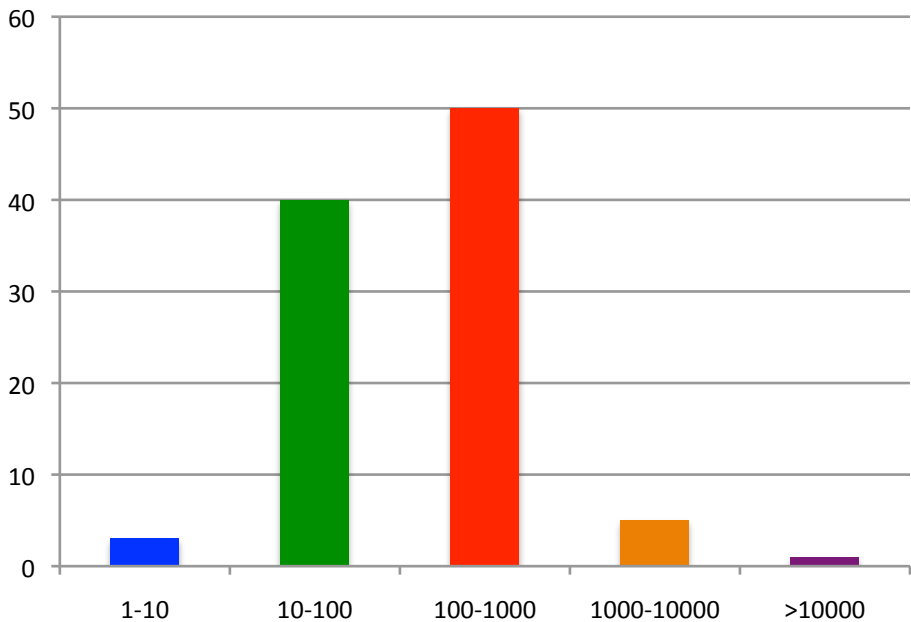

Supplement: Additional file 4 — Expression range. Abundance of annotated genes expressed between 1-10 FPKM, 10-100 FPKM, 100-1000 FPKM, 1000-10000 FPKM or more than 10000 FPKM. [file 1471-2164-12-552-S4.PDF]

a)

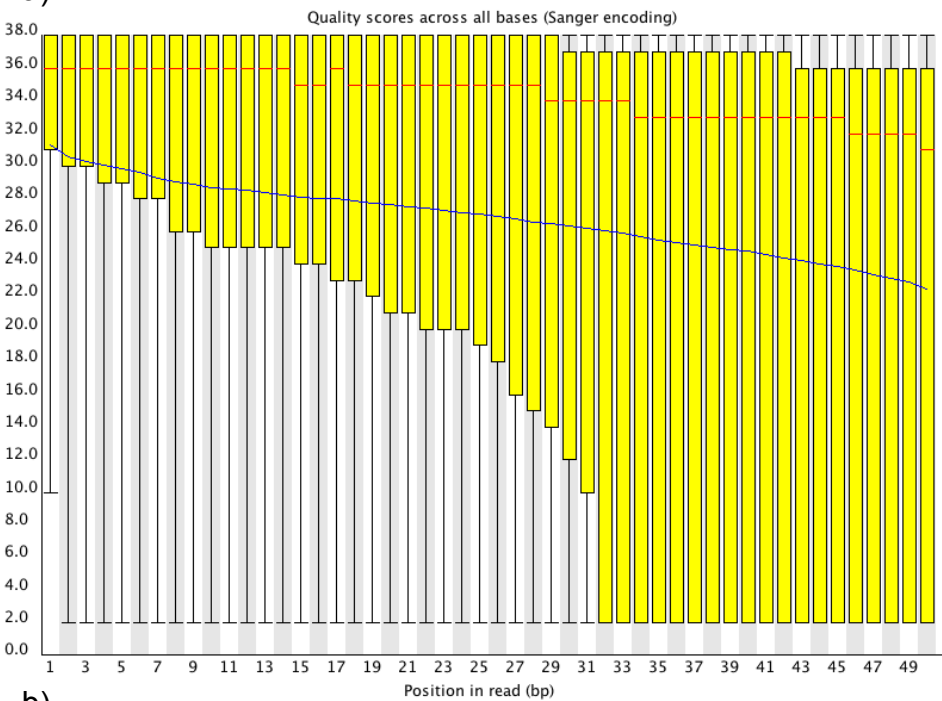

b)

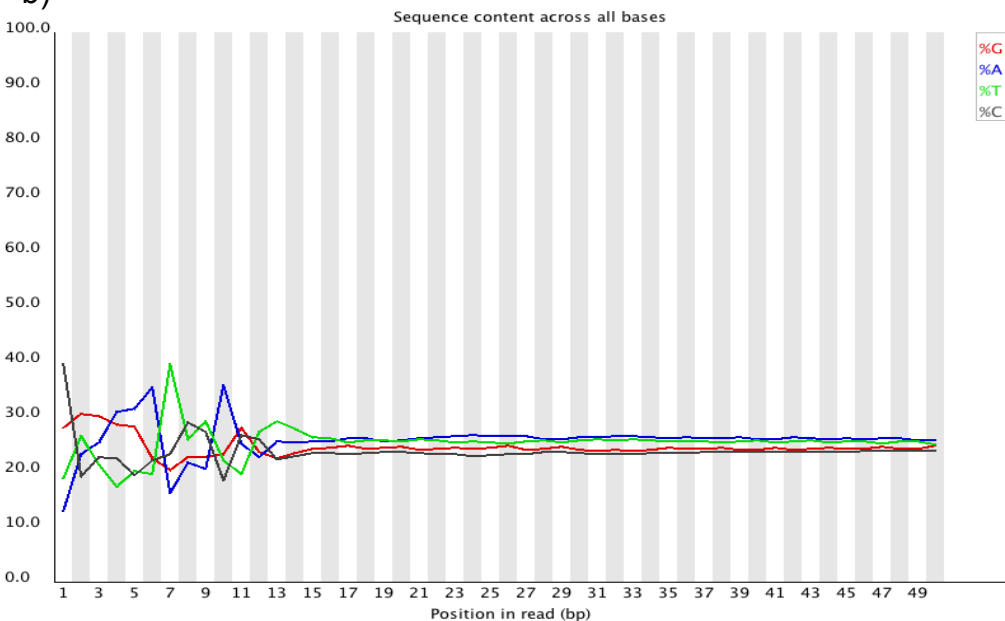

Supplement: Additional file 7 — Quality control of the reads. A) Raw reads quality control: Base qualities per cycle. B) Library sequencing bias: Proportion of bases incorporated in each sequencing cycle. [file 1471-2164-12-552-S7.PDF]
