# Supplementary material for: Exploring the gonad transcriptome of two extreme male pigs with RNA-seq
Source: BMC Genomics. 2011 Nov 8;12:552. doi: 10.1186/1471-2164-12-552 (PMC3221674; doi:10.1186/1471-2164-12-552)
Supplement: Additional file 6 — Sequence of the adapters. Where "P" refers to a PO4 moiety and * indicates a phosphorothioate bond. [file 1471-2164-12-552-S6.PDF]

**Large White**
PE_tagged_1a:<br>5'-P-GTTAGATCGGAAGAGCGGTTCAGCAGGAATGCCGAG-3'
PE_tagged_1b:<br>5'-ACACTCTTTCCCTACACGACGCTCTTCCGATCTAAC*T-3'
**Iberian**
PE_tagged_2a:<br>5'-P-CAAAGATCGGAAGAGCGGTTCAGCAGGAATGCCGAG-3'
PE_tagged_2b:<br>5'-ACACTCTTTCCCTACACGACGCTCTTCCGATCTTTG*T-3'
